# Estimating the Size of HIV Key Affected Populations in Chongqing, China, Using the Network Scale-Up Method

**DOI:** 10.1371/journal.pone.0071796

**Published:** 2013-08-13

**Authors:** Wei Guo, Shuilian Bao, Wen Lin, Guohui Wu, Wei Zhang, Wolfgang Hladik, Abu Abdul-Quader, Marc Bulterys, Serena Fuller, Lu Wang

**Affiliations:** 1 National Center for AIDS/STD Control and Prevention, Chinese Center for Disease Control and Prevention, Beijing, China; 2 U.S. Centers for Disease Control and Prevention, Global AIDS Program, Beijing Office, China; 3 Institute for AIDS/STD Control and Prevention, Chongqing Center for Disease Control and Prevention, Chongqing, China; 4 Division of Global HIV/AIDS, Center for Global Health, U.S. Centers for Disease Control and Prevention, Atlanta, United States of America; University of Ottawa, Canada

## Abstract

**Objectives:**

To estimate the average social network size in the general population and the size of HIV key affected populations (KAPs) in Chongqing municipality using the network scale-up method (NSUM).

**Methods:**

A general population survey was conducted in 2011 through a multistage random sampling method. Participants aged between 18 and 60 years were recruited. The average social network size (*c*) was estimated and adjusted by known population method. The size of HIV KAP in Chongqing municipality was estimated using the adjusted *c* value with adjustment for the transmission effect using the scaled respect factor.

**Results:**

3,026 inhabitants of Chongqing agreed to the survey, and 2,957 (97.7%) completed the questionnaire. The adjusted *c* value was 310. The estimated size of KAP was 28,418(95% Confidence Interval (*CI)*:26,636∼30,201) for female sex workers (FSW), 163,199(95%*CI*:156,490∼169,908) for clients of FSW, 37,959(95%*CI*: 34,888∼41,030) for drug users (DU), 14,975(95%*CI*:13,047∼16,904) for injecting drug users (IDU) and 16,767(95%*CI*:14,602∼18,932) for men who have sex with men (MSM). The ratio of clients to FSW was 5.74∶1, and IDU accounted for 39.5% of the DU population. The estimates suggest that FSW account for 0.37% of the female population aged 15–49 years in Chongqing, and clients of FSW and MSM represent 2.09% and 0.21% of the male population aged 15–49 years in the city, respectively.

**Conclusion:**

NSUM provides reasonable population size estimates for FSW, their clients, DU and IDU in Chongqing. However, it is likely to underestimate the population size of MSM even after adjusting for the transmission effect.

## Introduction

Chongqing, located in Southwest China, is the largest municipality in the country with over 28 million people living in 19 urban districts and 21 rural counties [1]. The first HIV case was reported in 1993. As of October 31, 2011, the cumulative number of reported HIV cases was 11,704. Majority of the cases reported to contract HIV infection through unsafe sexual behavior, with heterosexual transmission accounting for 49.3% of cases and homosexual transmission linked to 16.5% of cases. Injection drug use accounted for 23.7% of cumulative cases. From January through October 2011, 1,914 HIV cases were newly reported. Most of these cases were linked to either heterosexual (68.5%) or homosexual (18.4%) contacts [2]. With a large proportion of new HIV infections contributed to heterosexual contacts and a rapid increase in the number of HIV infected men who have sex with men (MSM), it is essential to understand the scope of persons who have been exposed to HIV associated high risk behavior in order to design targeted intervention programs and allocate limited resources more efficiently.

As most HIV key affected populations (KAPs) are hidden and hard to reach, Chongqing has limited data on the size of KAPs. There are no population size estimates for female sex workers (FSW) and men who have sex with men (MSM). The estimate available was 27,888 for drug users (DU) and 22,310 for injecting drug users (IDU), both derived in 2007 from the registered number of drug users at the local security department. Experts estimated the number of DU and IDU at 65,000 and 52,000, respectively [3]. However, KAP size in the city may have decreased as a result of enhanced crackdown against FSW and DU by public security bureaus since 2009. On the other hand, the increasing popularity of “night club drugs” such as amphetamine and methamphetamine in recent years would likely lead to an increase in the size of DU. In the meantime, HIV prevalence among MSM has increased rapidly [4]–[6] across the country. Chongqing municipality is one of cities in Southwest China where MSM population is heavily affected with HIV infection [7]. Given the possible changes in KAP size and distribution, it is important to generate new population size estimates. Conventional methods such as capture-recapture and multiplier require identification of and direct contact with hard to reach populations; therefore, the results are usually subject to sampling biases [8]. It is of great interest to explore new methods that can be implemented on a large-scale and may provide more reliable population size estimates.

This study used the network scale up method (NSUM) to estimate the size of KAP in Chongqing municipality. NSUM is a population-based survey method with the assumption that given a large sample size, the distribution of a subpopulation in the general population is similar to its distribution in the average social network of representative survey participants [9]. Therefore, the number of KAP members can be estimated based on the average size of the social network of the general population and the average number of KAP members known by survey participants. Compared with conventional methods commonly used for KAP size estimation, NSUM does not require people with risk behaviors to reveal their status to the investigators during interview, and no direct contact with KAP members is needed for the survey. Implementation of NSUM is also relatively easy as it can be integrated into other population surveys, and most importantly, it can give concurrent size estimates of multiple populations from a single survey [8]. In this study, NSUM was used to estimate the size of five key populations: FSW, clients of FSW, DU, IDU, and MSM in Chongqing.

## Methods

### Network Scale-up Method

The underlying assumption of NSUM is that people’s social networks are, on average, representative of the general population. Hence, in a representative sample of the general population, the proportion of KAP among the sample’s social networks can be approximated to the proportion of KAP in the general population [9], [10]–[11]. The formula is as below:
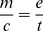
(1)


*m*: the number of people in a particular subgroup that the survey respondent knows.

*c*: the social network size of the survey respondent.

*e*: the population size of a particular subgroup.

*t*: the total number of the general population.

In this study, the “known population method” was used to estimate the *c* value [11]. Survey participants were asked about the number of people they knew in subpopulations of known size [12], from which the average *c* value was estimated using the maximum likelihood method as formula (2) [9] listed below:
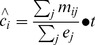
(2)



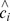
 :the social network size of respondent *i.*

*m_ij_*: the number of people in a particular subgroup *j* that the respondent *i* knows.

*e_j_*: the size of a particular subgroup *j.*

*t*: the total number of the general population.

The population size of a particular KAP was estimated using formula (3) [9] as below:
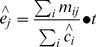
(3)


:the population size estimated.

*m_ij_*: the number of people in a particular subgroup *j* that the respondent knows.


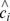
 :the social network size of respondent *i.*

*t*: the total number of the general population.

Standard error of the size estimate was calculated using formula (4) [9] as below:
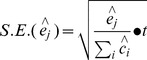
(4)


### Population Survey

The population-based survey was carried out between September and October 2011. Residents of Chongqing municipality aged between 18 and 60 years who had lived in the city for at least 6 months at the time of interview and who provided verbal informed consent to participate in the survey were recruited.

A multistage random sampling method was used to identify survey participants. Two (A and B) out of 19 urban districts and one (C) out of 21 rural counties were randomly selected. Within districts A and B, neighborhood, residential compounds, and residential buildings were the three stages in the sequential random sampling approach, in which a specific number of units within each stage were randomly selected. For county C, towns and villages were chosen using a similar random approach. A total of 3,000 households were then clustered sampled from selected residential buildings (District A and B) and villages (County C). One person per household was randomly selected for the interview after listing all the eligible persons within the household.

The anonymous self-administered survey was conducted with a structured questionnaire. Survey staff provided clarification when needed. The participants were asked the number of people they knew in 19 particular subgroups with known population size. They were also asked to report how many people they knew who were FSW, clients of FSW, DU, IDU, and MSM. All known population sizes came from National Bureau of Statistics, Chongqing Personnel Bureau, Chongqing Public Security Bureau, 2011 Statistical Yearbook, Chongqing Maternal and Child Center, Chongqing Disabled Federation and Chongqing Public Security Bureau, and detail information listed as below in table 1.

**Table 1:** Comparison between the official figure and estimated size of selected known populations after back estimation, Chongqing, China (2011).

Known population	Data sources(2010)	Statistics	Estimatednumber	Estimated/Statistics
**Males aged 20–24 years**	**National Bureau of Statistics**	**1,102,717**	**2,017,314**	**1.83**
**Females aged 20–24 years**	**National Bureau of Statistics**	**1,053,824**	**1,544,351**	**1.47**
**Females aged 70 years or older**	**National Bureau of Statistics**	**1,078,085**	**637,264**	**0.59**
Government officials	Chongqing Personnel Bureau	146,215	599,176	4.10
Registered policemen	Chongqing Public Security Bureau	29,891	222,465	7.44
Registered physicians	2011 Statistical Yearbook	44,844	447,360	9.98
Registered nurses	2011 Statistical Yearbook	37,462	236,286	6.31
Registered lawyers	2011 Statistical Yearbook	4,834	88,391	18.29
Registered primary or junior high school teachers	2011 Statistical Yearbook	193,146	701,939	3.63
Registered senior high school or college/universityteachers	2011 Statistical Yearbook	82,490	317,330	3.85
Junior high school students in 2010	2011 Statistical Yearbook	1,281,724	511,644	0.40
**Senior high school students in 2010**	**2011 Statistical Yearbook**	**626,434**	**371,164**	**0.59**
People who were divorced in 2010	2011 Statistical Yearbook	190,104	83,185	0.44
**People who were married in 2010**	**2011 Statistical Yearbook**	**626,786**	**371,077**	**0.59**
People who were injured or died in a caraccident in 2010	2011 Statistical Yearbook	9,804	69,107	7.05
**People who died in 2010**	**2011 Statistical Yearbook**	**185,500**	**185,967**	**1.00**
**Women who gave birth in 2010**	**Chongqing Maternal and Child Care Center**	**294,264**	**210,906**	**0.72**
Handicapped people in 2010	Chongqing Disabled Federation	1,265,200	176,142	0.14
**People in detention/jail in 2010**	**Chongqing Public Security Bureau**	**55,200**	**28,135**	**0.51**

**Bold: populations kept after back estimation.**

The working definition of “people whom the participant knows” was an individual 1) who has lived in Chongqing municipality for at least 6 months, 2) whom the participant has met in person before, 3) who knows the participant by sight or name and vice versa, and 4) whom the participant has contacted in the past 2 years, including through in-person visits, phone calls, or e-mail.

The working definitions of FSW, clients, DU, IDU, MSM were among the persons whom the participant knows, the individuals who had high risk behaviors in the last two years. FSW and its clients were defined as person who have heterosexual behaviors in exchange for money; MSM was a man have had sex with another man regardless sexual orientation; and DU/IDU were the users of heroin, opium, opiate analgesics, cocaine, marijuana, methamphetamine, ketamine, methylene dioxymetham-phetamine [MDMA], and other newer so called “club drugs”, by any means, including injection or non-injection.

### Ethics Statement

The protocol, informed consent form, and questionnaire were reviewed and approved by the Institutional Review Board (IRB) of the National Center for AIDS/STD Control and Prevention (NCAIDS), Chinese Center for Disease Control and Prevention (China CDC), and the Division of Global HIV/AIDS, Center for Global Health, U.S. CDC. Verbal informed consent was obtained from each participant prior to their participation.

### Estimation of Social Network Size (*c* Value)

According to formula (2) and (3), *m*_i_ is essential for estimating *c* value and the size of KAPs. In order to have a representative *m_i_* for both known populations and KAPs, *m_i_* were firstly weighted by demographic distribution before calculating *c* value for general population and estimation KAPs size, so that the respondents have the same gender (male and female), age groups (∼19, 20∼29, 30∼39, 40∼49,50∼), and education level (‘High school or below’ and ‘Junior college or above’) proportion as the general population from 2010 population census data in Chongqing. A weight was calculated that is the proportion of a sub-population with specific demographic characteristics among general population divided by the proportion of that kind of sub-population among our surveyed respondents.

The *c* value was estimated using the maximum likelihood method (Formula 2). Three steps were taken to adjust the *c* value. Firstly, the *c* value was calculated with weighted *m_i_* for known population, in equation (2). Secondly, the back estimation method was applied to identify those known populations that generated less biased estimates of *c*. The nineteen subgroups with known population size used for initial analysis included individuals in certain gender and age groups, people who had specific occupations (such as governmental officials, policemen, physicians, nurses, high school teachers, and students), those with recent changes in marital status such as newly married and divorced in 2010, persons in custody, and those who died in 2010. With back estimation method, the population size of each known population was estimated using the average *c* value generated by the rest of the 18 known populations. A reality check was performed to compare the ratio of the estimated size to the known statistical data [10]. A ratio of less than 0.5 or over 2.0 indicated that the estimate of the known population was at least 50% underestimated or 100% overestimated, respectively. Such groups were deemed unsuitable for use in data analysis and excluded from estimating *c*. Lastly, *c* values were log transformed and outliers of *c* value beyond 1.5 times of quartile range were excluded, that is, *c* value which was less than P_25_ minus 1.5 times of quartile range or larger than P_75_ plus 1.5 times of quartile range was deemed as outlier and was excluded.

As the *c* values did not follow a normal distribution, the Kruskal-Wallis test was used to compare the *c* value of people with different demographic characteristics.

### Estimation of KAPs Sizes with Social Respect Adjustment

Population sizes and 95% confidence interval were derived using formula 3 and 4. KAPs sizes tend to be underestimated due to transmission and barrier effects and estimation effect [12], [13]. The transmission effect occurs when the risk behavior information about a person is not transmitted with the same probability within that person’s social network. When some social and/or geographical barriers keep people from knowing specific groups of the population, there is the barrier effect and when respondents cannot recall accurately about the number of people belonging to a particular subpopulation, the estimation effect occurs [11], [12]. To account for the social desirability bias, besides the demographic adjustment on *m_i_*, a social respect factor was used for adjustment additionally. The respondents were asked to rank their respect for each KAP on a scale of 1 = very low to 5 = very high, among which the scales of 2, 3, 4 represent a respect level of low, medium, and high, respectively. In this study, disrespect for KAP was defined as having a scale value of very low and low. For each KAP, the knowing number of KAP to respondents was weighted with a factor of *Wri*, which was used to reflect the impact of respect level on knowing KAP among respondents. *Wr_i_* was defined as the average number of the target population known to respondents with a respect level *i* (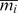) divided by average number of the target population known to the respondents with a medium level of respect (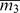). With the introduction of *Wri*, equation (3) was transformed to equation (5) as below during the estimation KAP population size:
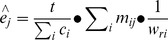
(5)


For example, if the average number of FSW known to the respondents with respect level *i* is 0.5 (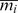 *)* and 0.8 (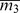 ) for those with a medium respect level of 3, then the factor *Wri = *0.5/0.8 = 0.625 was used to reflect the impact of respect level on knowing FSW. During the estimation of KAP size(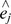), *Wr_i_* will be used to adjust the number of FSW (*m_ij_*) for each respondents with respect level to FSW in equation (5). This assumes that a person who holds a neutral opinion on a specific KAP is least likely to over or under report the number of people known in that population. For DU/IDU, as the question for respect level was designed only for DU; therefore, we used the same social respect factor for DU and IDU.

According to the HIV integrated biological and behavioral surveillance data in Chongqing over the past three years, the majority of the KAP populations was aged between 15 and 49 years old (98% of FSW, 90% of MSM, 97% of DU, and 85% of male attendees of STD clinics (a proxy for the clients of FSW)). Therefore, to calculate the percentage of a specific KAP in general population, the estimated population size after adjustment with respect factor was divided by the size of female (if FSW) or male (if clients of FSW or MSM) population aged 15–49 years in Chongqing from census data.

All data analysis was performed using IBM SPSS Statistics 18.0.

## Results

### Characteristics of Survey Participants

In total, 3,026 questionnaires were obtained, and 2,957 of them were valid for further analysis. Of these, 998 (33.8%) valid records were collected from county C, 1,313 (44.4%) from district B, and 646 (21.8%) from district A. In district A and B, 3,635 households were reached. Among these, 1,373 (37.8%) households were either vacant during two visits or all the persons in the household were older than 60 years. Another 244 (6.7%) eligible residents refused to participate. In county C, 1,008 questionnaires were collected in three main streets household by household.

Most respondents (60.3%) were female. The mean age was 39.2±11.5 years. Over half of the participants (56.6%) had an educational level of junior or senior high school, and 82.4% were married. Most participants (82.1%) had lived in Chongqing municipality for more than 3 years. The demographic features of survey participants are listed in Table 2. The demographic distribution was significantly different for gender (*χ^2^* = 141.66, *P*<0.0001), age groups (*χ^2^* = 32.88, *P*<0.0001), education level (*χ^2^* = 177.94, *P*<0.0001) compared to the 2010 census data for Chongqing.

**Table 2:** Average social network size by demographic characteristics, Chongqing, China (2011).

Demographic characteristics	Respondents		Social network size (c)		
	Number	%	Crude	*P* value*	Adjusted
**District/County**					
District A	646	21.8	326	<0.0001	272
District B	1,313	44.4	280		244
County C	998	33.8	397		425
**Gender**					
Male	1,173	39.7	383	<0.0001	446
Female	1,784	60.3	295		223
**Age**					
18∼19	71	2.4	552	<0.0001	943
20∼29	630	21.3	487		363
30∼39	833	28.2	331		304
40∼49	769	26	293		297
50∼60	654	22.1	195		219
**Education**					
Illiteracy	116	3.9	159	<0.0001	160
Primary school	516	17.5	204		225
Middle school	919	31.1	251		285
High school or technical secondary school	756	25.6	300		363
Junior college or above	650	22	605		384
**Marital status**					
Cohabitation	36	1.2	610	<0.0001	453
Married	2,437	82.4	299		288
Single	391	13.2	512		459
Divorced or widowed	93	3.1	249		234
**Residence**					
6–12 months	140	4.7	298	0.008	260
1–3 years	387	13.1	320		273
More than 3 years	2,430	82.2	333		320
Total	2,957	100.0	330		311

* Kruskal-Wallis test.

### Estimation of Social Network Size and Adjustment

The estimated crude average social network size (*c* value) was 330 (standard deviation [SD] 367, median 208).There was significant differences in *c* by demographic characteristic (Table 1). Inhabitants in rural area (residents in county C) had the largest social networks compared to those in urban areas (residents in district A and B) (*χ* ^2^ = 146.17, *P*<0.0001); males tended to know more people than females (Z = −7.39, *P*<0.0001); younger people were more socially active than older people (*χ* ^2^ = 339.66, *P*<0.0001); people with higher education appeared to have larger networks than those with lower education (*χ* ^2^ = 469.67,*P*<0.0001); and unmarried persons generally had larger social networks than married persons (*χ* ^2^ = 180.80, *P*<0.0001).

After adjustment for the demographic distribution of gender, age group, and education level, the average *c* value was 311 with a SD of 360 and a median of 195.

Using the exclusion criteria of a ratio outside the range of 0.5 and 2.0, 11 known populations were excluded, leaving 8 known populations to generate a final *c* value of 311 with a SD of 370 and a median value of 195 (Table 2).

**Figure 1:**
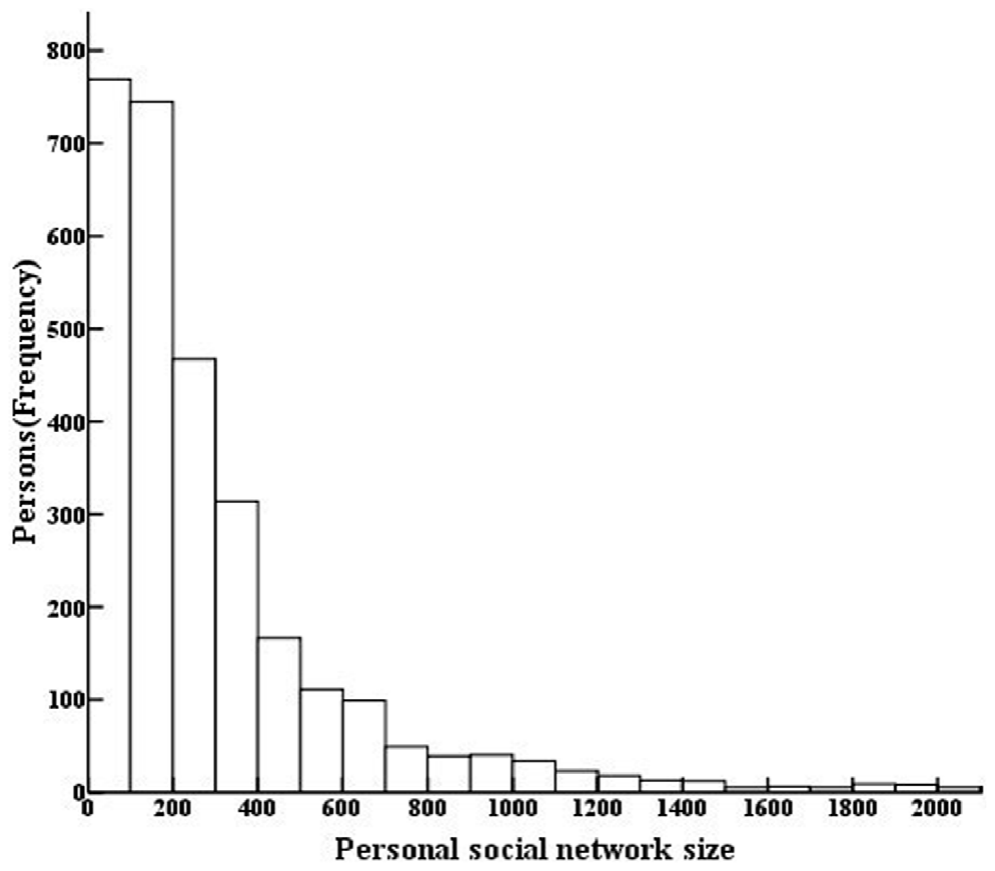
Distribution of social network size, Chongqing, China (2011). (Instances of *c* more than 2,000 are not shown directly in Figure 1.).

The distribution of *c* was positively skewed (Figure 1). Following log transformation, outliers beyond 1.5 times of quartile range were excluded. The resulting Log (*c*) values were normally distributed (*P*>0.05). After exclusion of outliers, the average *c* value was 310 with a SD of 341 and a median of 199.

### Estimation of KAPs Sizes and Adjustment for Transmission Effect

1. Knowledge of KAP: Of 2,957 participants, 229 (7.7%) reported that they knew FSW, 480 (16.2%) knew clients of FSW, 262 (8.9%) knew DU, 113 (3.8%) knew IDU, and 113 (3.8%) knew MSM (Table 3). The proportion of people who knew persons from the specific KAP varied significantly by location; more people knew FSW in county C whereas more people knew DU and IDU in district B. Overall, people living in districts A and B tended to know more MSM than those in county C (Table3).
2. Adjustment with social respect: According to the response of respect level for KAP, over 60% of the participants viewed KAP with low or very low respect. IDU/DU received the lowest respect with a disrespect rate of 90.3% (2,669/2,957). Data also showed that as respect level increased, respondents knew more people in KAP, except for FSW (Table 4). Based on the average number of acquaintances in KAP and respect levels, social respect factors for adjusting population size estimates were calculated. The social respect factor was 0.90 for FSW, 2.22 for their clients, 2.00 for DU/IDU, and 2.25 for MSM.
3. Estimation of KAPs sizes: KAPs sizes were estimates after the adjustment of c value. The estimated population size of FSW was 31,576(95%*CI*: 29,595∼33,556), for clients of FSW 73,513(95%*CI*:70,491∼76,535), for DU 18,979(95%*CI*:17,444∼20,515), for IDU 7,488(95%*CI*:6,523∼8,452), and for MSM 7,452(95%*CI*:6,490∼8,414). Adjustment with the respect factor increased these values for all groups except FSW (Table 5). After adjustment, the estimated size was 28,418(95%*CI*:26,636∼30,201) for FSW, 163,199(95%*CI*:156,490∼169,908) for clients of FSW, 37,959(95%*CI*:34,888∼41,030) for DU, 14,975(95%*CI*:13,047∼16,904) for IDU and 16,767(95%*CI*:14,602∼18,932) for MSM. The ratio of clients to FSW was 5.74∶1, and IDU accounted for 39.5% of the DU population. These estimates suggest that FSW account for 0.37% of the female population aged 15–49 years in Chongqing, and clients of FSW and MSM represent 2.09% and 0.21% of the male population aged 15–49 years in the city, respectively.

**Table 3:** The number of respondents who knew KAP, by district/county, Chongqing, China (2011).

KAP	Total (n = 2957)		A district (n = 646)		B district (n = 1,313)		C county (n = 998)		?2	*P* value
	Number	%	Number	%	Number	%	Number	%		
FSW	229	7.7	7	1.1	48	3.7	174	17.4	202.01	<0.0001
Clients	480	16.2	123	19	184	14	173	17.3	9.39	0.009
DU	262	8.9	44	6.8	169	12.9	49	4.9	48.81	<0.0001
IDU	113	3.8	15	2.3	75	5.7	23	2.3	22.97	<0.0001
MSM	115	3.9	32	4.9	57	4.3	26	2.6	7.08	0.029

**Table 4:** The average number of acquaintances in KAP by respect level, Chongqing, China (2011).

Respect level	FSW		DU/IDU**		Clients		MSM	
	Acquaintances^*^	N	Acquaintances	N	Acquaintances	N	Acquaintances	N
Very High	0.50	4	0	2	3.33	3	0.33	6
High	0.40	164	0.25	36	1.75	69	0.38	187
Medium	0.27	757	0.40	250	1.64	711	0.18	703
Low	0.26	1693	0.20	1629	0.43	1719	0.03	1692
Very Low	0.47	339	0.16	1040	0.36	455	0.02	369
Total	0.30	2957	0.20	2957	0.74	2957	0.08	2957

Note: *Acquaintances in the table above denote the average number of acquaintances in KAP.

** The same social respect factor was used for DU and IDU.

**Table 5:** The estimated number of people in KAP, Chongqing, China (2011).

KAP	Without Correction	Adjusting factor	With Correction
	Point estimate(95% *CI*)		Point estimate(95% *CI*)
FSW	31,576(29,595∼33,556)	0.90	28,418(26,636∼30,201)
Clients	73,513(70,491∼76,535)	2.22	163,199(156,490∼169,908)
DU	18,979(17,444∼20,515)	2.00	37,959(34,888∼41,030)
IDU	7,488(6,523∼8,452)	2.00	14,975(13,047∼16,904)
MSM	7,452(6,490∼8,414)	2.25	16,767(14,602∼18,932)

## Discussion

This study facilitated the simultaneous estimation of all key affected population sizes in Chongqing municipality, providing new and updated estimates that are urgently needed for local HIV control efforts. To date, the only available population size estimate had been for DU and IDU [3]. KAPs sizes are important for understanding local concentrated HIV epidemics and for improving program planning. However, it is often difficult to obtain reliable population size estimates due to widespread stigma and discrimination against these populations [14].

The average social network size (*c*) is a key parameter for NSUM. Various approaches have been used to adjust the crude *c* value. Salganik *et al*. limited the maximum number of people that survey participants could report to know in each known population at 30 [15].Our study used 99 as the maximum number, which may lead to a larger social network size estimate. A study in Ukraine [12] withdrew about 5% of maximum reported *c* values and then selected known populations using the back estimation method. Research in Italy excluded some known populations using residual plot by comparing the association between the estimated population size and the statistics [12]. In Japan, data were adjusted to reflect the known distribution of gender and age groups in the general population; then the back estimation method was used to evaluate the reliability of the estimates of known populations [16]. Our study used a three-step approach to estimate *c*. First, the data were weighted by gender, age group, and education level based on the 2010 census data. Then the back estimation method was applied to exclude known populations that led to extreme over or underestimation. Lastly, *c* values were log transformed and outliers were eliminated from further estimation.

Our study’s *c* values showed a positively skewed distribution among the general population in Chongqing municipality, similar to those seen in the U.S. and Brazil [9], [15]. The known population method of this study yielded an average social network size of 310, comparable to the size of 290 in the U.S. where both known population and summation methods were used [11]. Our study found that social network size varies by location, gender, age, and education level. Rural residents tended to have larger networks than urban residents. Larger social network values were also found among males, young people, and people with higher education levels.

NSUM builds upon the assumptions that everyone in the general public has an equal chance of knowing KAP members and everyone has perfect knowledge about their acquaintances [9]–[10]. However, these assumptions rarely hold due to stigma and discrimination, thus the results are generally biased. Some methods have been explored to account for such biases. Researchers in Ukraine [13] and Moldova [17] adjusted the results using a scaled social respect factor. In Brazil, a game-like survey involving playing cards and a game board (‘game of contacts’) was performed among heavy drug users [18] to estimate the information transmission rate and potential differences in social network sizes between heavy drug users and the general population. In Japan, researchers used the proportion of MSM who had ever disclosed their sexual orientation to acquaintances (come out rate) to adjust for transmission effects [16]. Our study used the social respect factor similar to Ukraine and Moldova to adjust population size estimates. However, during the estimation of FSW population, the overall respect factor for FSW was 0.9, that means that respondents were willing to know FSW. This bias was mainly caused by the special situation in County C, where is a famous resorting place in Chongqing, and people living there with disrespect attitude to FSW also have more chance to know FSW.

In our study, clients of FSW are estimated to account for approximately 2% of males aged 15–49 years, whereas FSW account for 0.4% of all females aged 15–49 years, resulting in a ratio (clients to sex workers) of 5.74∶1. These estimates are close to the values recommended in the national HIV epidemic estimate guidelines where the proportion of FSW among females aged 15–49 years is between 0.2% and 0.8%, and the ratio of clients to FSW is between 5∶1 and 10∶1 [19]. A study in Zhejiang Province in China estimated the ratio between 6.5∶1 and 11.8∶1 [20]. The proportion of FSW in female population in our study is close to those reported in India, Nepal, and Indonesia [21], and the proportion of clients in male population is similar to estimates in the Philippines [22]. Our study also found the proportion of IDU among all DU to be about 40%, which is lower than 61.2% from national HIV sentinel surveillance [23].

According to this study, only 0.21% of adult males aged 15–49 years in Chongqing municipality are engaged in homosexual behaviors. This is much smaller than those obtained by the multiplier method elsewhere in China. A study in Shanghai municipality [24] indicated a proportion of 6.6 ∼ 7.1% among adult males aged 15–49 years were MSM. Studies in Beijing and Harbin showed the proportion of MSM to be 1.0% and 0.3% [25], with the latter likely underestimated due to sampling bias. High degree of social stigma and discrimination against MSM in China may have a strong impact on the reliability of the estimate in our study. Many MSM only identify their sexual orientation to intimate acquaintances. The study in Japan noted that, on average, only 5.1 acquaintances of MSM knew their status [14]. It will be very hard to obtain an accurate population size estimate without adequate adjustment for the transmission error. Similar to the results of our study, the study in Ukraine suggested an underestimation of MSM population size despite adjustment with the social respect factor [11]. The Japan study adjusted the result by using “come out rate” directly, which gave an estimate of 2.87% in study areas [16].Interestingly, one recent study in Iran compared two approaches of NSUM for estimating population sizes. The results derived from the probability method in which people were asked whether they knew anybody in a specific subpopulation were closer to the external data and were generally greater than those derived from the frequency method in which people were asked to report the number of acquaintances in a particular subpopulation. The study showed that the proportion of MSM was 2.5% based on the probability method and 0.5% based on the frequency method [26].

Notably, a more strict definition of acquaintance was used in this study, that is, survey participants had to have met the acquaintance in person before. The definition commonly used in other studies [9]–[12], [14]–[15]was “People whom you know and who know you, in appearance OR by name, with whom you can interact, if needed, and with whom you have contacted over last two years personally or by telephone, or e-mail”, which would include acquaintances the participants may have never had personal contact with. The definition we used may yield a smaller average *c* value, and thus may lead to an overestimation of the population size. This degree of overestimation needs to be determined by further investigation.

There are some limitations to this study. First, obtaining a representative sample is challenging in Chongqing municipality, which has over 28 million residents. To account for discrepancies in demographic distributions between survey participants and census data, post-stratification measures were taken to adjust for the distribution of key demographic characteristics. Second, survey participants may have had different interpretations of the definitions of key populations and acquaintances. This may affect the accuracy of the reported information. Thirdly, the study suffered from the inherent biases of NSUM due to transmission effect, barrier effect, and estimation effect [8], [11]. In this study, a scaled social respect factor was used to adjust for the likelihood that the survey participants hesitate to report that they know someone in the key populations. However, this adjustment may have accounted for only some of the biases present. It did not directly address the errors that occurred when the respondents were not fully aware of the behaviors of the members of their personal network. Inadequate adjustment for transmission effect will produce an underestimate of KAP size. Adjustment with social respect factor is also likely to introduce more uncertainty to the estimates and the width of confidence interval will be enlarged. Additionally, of the best knowledge of the authors, no suitable adjustment methods have been developed yet to account for the biases due to barrier effects. Lastly, this survey ignored the social network differences between KAP and the general population comparing KAP to that of the general population [15]. Using a generalized network scale up method, which adjusted for both transmission effect and differential network size, Salganik et al obtained a population size as two times large as the estimate of NSUM [15].

This is the first study in China using NSUM to estimate the size of HIV KAPs. The study verified the feasibility of this method in China. It produced estimates for FSW, clients of FSW, DU, and IDU consistent with national guidance; however, it yielded a low MSM population size estimate.

No gold standard for key affected population size estimation is currently available as all known methods are subject to their own limitations. While NSUM is still under evaluation in the field of HIV key affected populations, this method represents a welcome addition to existing techniques.
